# Extracellular vesicle mediated intercellular communication at the porcine maternal-fetal interface: A new paradigm for conceptus-endometrial cross-talk

**DOI:** 10.1038/srep40476

**Published:** 2017-01-12

**Authors:** Mallikarjun Bidarimath, Kasra Khalaj, Rami T. Kridli, Frederick W. K. Kan, Madhuri Koti, Chandrakant Tayade

**Affiliations:** 1 Department of Biomedical and Molecular Sciences, Queen’s University, Kingston, Ontario, K7L 3N6, Canada; 2 Department of Biomedical Sciences, Ontario Veterinary College, University of Guelph, Guelph, Ontario, N1G 2W1, Canada; 3 Department of Animal Production, Faculty of Agriculture, Jordan University of Science and Technology, Irbid, 22110, Jordan

## Abstract

Exosomes and microvesicles are extracellular vesicles released from cells and can contain lipids, miRNAs and proteins that affect cells at distant sites. Recently, microvesicles containing miRNA have been implicated in uterine microenvironment of pigs, a species with unique epitheliochorial (non-invasive) placentation. Here we report a novel role of conceptus-derived exosomes/microvesicles (hereafter referred to as extracellular vesicles; EVs) in embryo-endometrial cross-talk. We also demonstrate the stimulatory effects of EVs (PTr2-Exo) derived from porcine trophectoderm-cells on various biological processes including the proliferation of maternal endothelial cells (PAOEC), potentially promoting angiogenesis. Transmission immuno-electron microscopy confirmed the presence of EVs in tissue biopsies, PTr2-Exo and PAOEC-derived EVs (PAOEC-Exo). RT-PCR detected 14 select miRNAs in CD63 positive EVs in which miR-126-5P, miR-296-5P, miR-16, and miR-17-5P were the most abundant angiogenic miRNAs. Proteomic analysis revealed EV proteins that play a role in angiogenesis. *In-vitro* experiments, using two representative cell lines of maternal-fetal interface, demonstrated bidirectional EVs shuttling between PTr2 and PAOEC cells. Importantly, these studies support the idea that PTr2-Exo and PAOEC-Exo containing select miRNAs and proteins can be successfully delivered to recipient cells and that they may have a biological role in conceptus-endometrial cross-talk crucial for the pregnancy success.

Exosomes are membrane-bound bioactive nanovesicles (30–100 nm) of multivesicular body origin that can be released from the cell surface by exocytosis^1^,^2^. Most cell types secrete exosomes and often reflect aspects of the physiological state and function of the originating cells, including the placenta and endometrium^3^,^4^,^5^. Exosomes/microvesicles (hereafter referred to as extracellular vesicles; EVs) can reach bodily fluids, such as plasma^6^, urine^7^, amniotic fluid^8^, semen^9^, milk^10^, saliva^11^ as well as uterine luminal fluid in sheep^12^ and pigs^13^. In addition, the release and content of the EVs can be influenced by the extracellular micro-environment^14^. The exosomal lipid bilayer made up of relatively high concentrations of cholesterol, sphingomyelin, ceramide and detergent resistant membrane domains making these vesicles very stable in extracellular space^15^. In addition, exosomes possess surface receptors/ligands of the original cells and have the potential to selectively interact with specific target cells^16^. Intracellular pathways can also be regulated by the exosomes which can sequester signaling molecules in the cytoplasm either by reducing their bioavailability or preventing their packaging and release via exocytosis^17^. Reports to date provide evidence that exosomes contain lipids, proteins, mRNA and numerous small non-coding RNAs (~22 nucleotides) such as miRNAs^1^,^5^,^18^,^19^. Exosomes can horizontally transfer mRNA and miRNAs to other cells. mRNAs can then be translated into functional proteins in the new location and miRNAs can exert gene silencing in the recipient cells^20^,^21^. For instance, exosomes can elicit biological effects, such as increased endothelial cell proliferation and migration at the implantation site^22^,^23^, that are important for conceptus development in pigs.

Embryo implantation in pigs is a complex process that requires a synchronized reciprocal dialogue between a receptive endometrium and developing blastocysts^24^,^25^. During the early implantation period in pigs (Days 4–15 of pregnancy), the developing conceptus (including embryo, trophectoderm and associated extra-embryonic membranes) undergoes rapid morphologic changes (from spherical to tubular to filamentous forms) and migrates freely through the entire lumen of the uterus^24^. During days 15–20 of pregnancy, the initiation of porcine placentation is characterized by the expression of a unique repertoire of adhesive molecules on the surface of both the trophectoderm and the uterine luminal epithelium enabling the firm attachment^25^. This follows the dramatic change in physiological processes including angiogenesis on the endometrial side^26^. Unlike humans and mice, placental tissues in pigs do not invade the endometrium but, instead, lay in close apposition leading to the establishment of non-invasive epitheliochorial placenta by days 26–30 of pregnancy^27^,^28^. Once established, adhered trophoblast-endometrial epithelial bilayer initiates to develop firm contact in order to facilitate nutrient exchange across maternal-fetal interface^29^,^30^.

Cellular communication at the maternal-fetal interface during early gestation is crucial and thus determines the fate of pregnancy. Successful placentation allows rapid exchange of biomolecules between the endometrium and the developing conceptus^31^. The growing conceptus has to communicate with endometrium via angiogenic signals in order to get enough supply of nutrients through developing vasculature. In return, cells of the uterine microenvironment could also send signals to trophectoderm to influence the growth of conceptus^25^,^32^. Two decades ago, electron microscopic studies of porcine fetal-maternal interface revealed abundant secretory vesicles that were distributed over the microvilli during the peri-implantation period^33^. However, it was not clearly known until the discovery of exosomes that they provide an alternate mode of cell-cell communication. It is now well established that exosomes secreted by placental cells can cross the maternal side to influence biological functions of the recipient cells^4^,^22^,^34^. However, there is a dearth of information on how exosomes containing numerous biomolecules including miRNAs can migrate bi-directionally to modulate the pregnancy related processes including angiogenesis in pigs. We hypothesize that EVs secreted by trophectoderm can be internalized by the endothelial cells of the developing vasculature of the endometrium and vice-versa.

We examined porcine endometrium and the chorioallantoic membrane (CAM) isolated at day 20 of pregnancy, as well as porcine trophectoderm cells (PTr2) for EV membrane marker protein, CD63. We then harvested and positively identified EVs released by endometrial and CAM tissues as well as PTr2 and porcine aortic endothelial cells (PAOEC) using CD63 marker and size analysis. Further, we profiled 14 selected miRNAs present in PTr2 and PAOEC cells and their EVs. Mass spectrometry based analysis identified the proteins present in the EVs and bioinformatically studied their implications in signaling pathways relevant to early events in porcine pregnancy. Importantly, we demonstrated the shuttling of PTr2-Exo into PAOECs and vice-versa. Finally, we assessed the effect of PTr2-Exo on endothelial cell proliferation and vice-versa. The present study provides novel insights into the current understanding of embryo-endometrial communication in a unique non-invasive placental type seen in pigs.

## Results

### Extracellular vesicles are present and secreted by the porcine endometrium as well as chorioallantoic membrane

**Figure 1:**
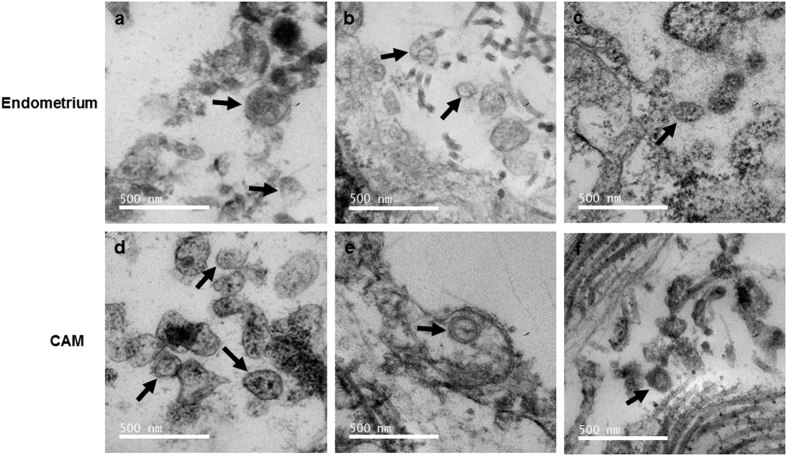
Extracellular vesicles released by porcine endometrium and chorioallantoic membrane (CAM) were identified by transmission electron microscopy (TEM) on representative ultrathin sections. (**a**–**f**) Endometrial and CAM biopsies were isolated from the conceptus attachment site at gestation day 20. TEM revealed vesicles of size in the range of approximately 50–150 nm, consistent with EVs in both the endometrium and CAM. Endometrial EVs (black arrows) appear to be localized in the extracellular space (**a–c**) while EVs (black arrows) in CAM are localized in the close proximity of cell membrane (**d–f**). Data is derived from three independent experiments. Scale bar: 500 nm.

We first investigated whether porcine endometrium and CAM could release EVs into the extracellular space. As shown in the Fig. 1a–c, transmission electron microscopy revealed the presence of EVs in the extracellular space of endometrium. Similarly, ultrathin sections of CAM (Fig. 1d–f) revealed the presence of EVs lying in close proximity to the cell membrane with a round shaped morphology and presence of EV membrane. Most EVs were in the 36- to 147- nm range.

### Characterization of extracellular vesicles derived from PTr2 and PAOEC cells

**Figure 2:**
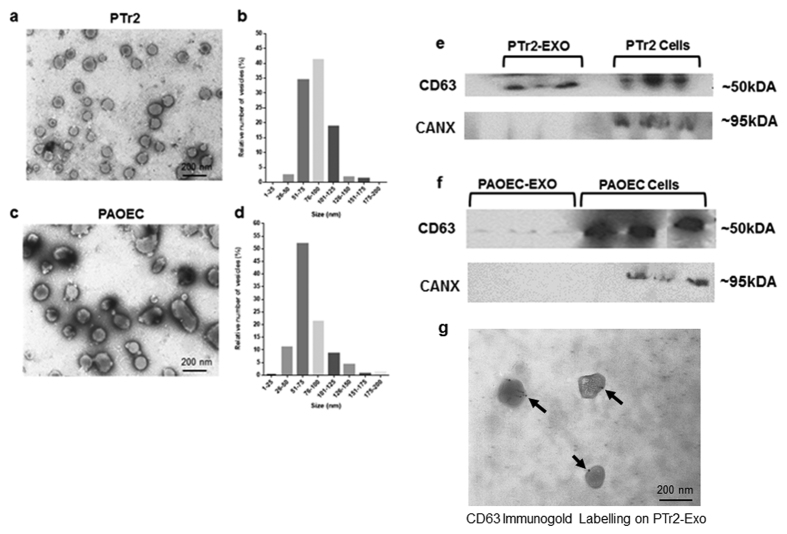
Characterization of EVs isolated from culture supernatants of PTr2 and PAOEC cells. (**a**) Transmission electron microscopy (TEM) of PTr2 derived EV pellets that are negatively stained with uranyl acetate and lead citrate. (**b**) Histogram of the number of isolated PTr2 derived EVs diameters. The Y axis shows the relative number of vesicles (%), and the × axis shows the vesicle diameter (nm). The size of EVs was approximately in the range of 26- to 125- nm (diameter [mean ± SD], 86 ± 21 nm). (**c**) TEM analysis of PAOEC derived EVs. (**d**) PAOEC derived EVs measured approximately in the range of 26- to 150- nm (diameter [mean ± SD], 99 ± 26 nm). (**e**) Western blotting detected CD63, exosomal marker, in the EV fraction as well as cellular fraction derived from both the PTr2 and PAOEC (**f**; cropped blots are displayed), respectively. (See also full-length blots in the Supplementary Figure S1). Calnexin (CANX) was only detected in cell lysates of PTr2 and PAOEC cells. (**g**) Characterization of EVs isolated from culture supernatant of PTr2 cells using transmission immunoelectron microscopy. Negatively stained EVs are labelled with 12-nm colloidal gold particles that recognize CD63 (black arrows) on the exosomal membrane. Data is derived from three independent experiments. Scale bar = 200 nm.

To determine whether PTr2 and PAOEC cells can secrete EVs, we isolated EVs from the cell supernatants and examined these via transmission electron microscopy. The diameter of EVs released by PTr2 (Fig. 2a) was found to be in the range of 26- to 125- nm with an average diameter of 86 ± 21 nm (Fig. 2b) and the diameter of EVs from PAOEC (Fig. 2c) was in the range of 26- to 150- nm with an average diameter of 99 ± 26 nm (Fig. 2d). Because EVs are known to express CD63, a well characterized exosome protein marker^35^, we performed western blotting for CD63. As shown in Fig. 2e,f, Western blotting detected CD63 in the EV fraction as well as cellular fraction derived from both the PTr2 and PAOEC, respectively (See also Supplementary Fig. S1 for blots). An endoplasmic reticular protein, Calnexin (Negative control) was absent in the EV preparation while it was detected in the cell lysates. Furthermore, we also performed immunolabelling on PTr2 derived EVs. Morphometric analysis of isolated EVs showed that CD63 is expressed on the membrane (Fig. 2g).

### Porcine endometrium, chorioallantoic membrane and PTr2 cells express CD63, exosome protein marker

**Figure 3:**
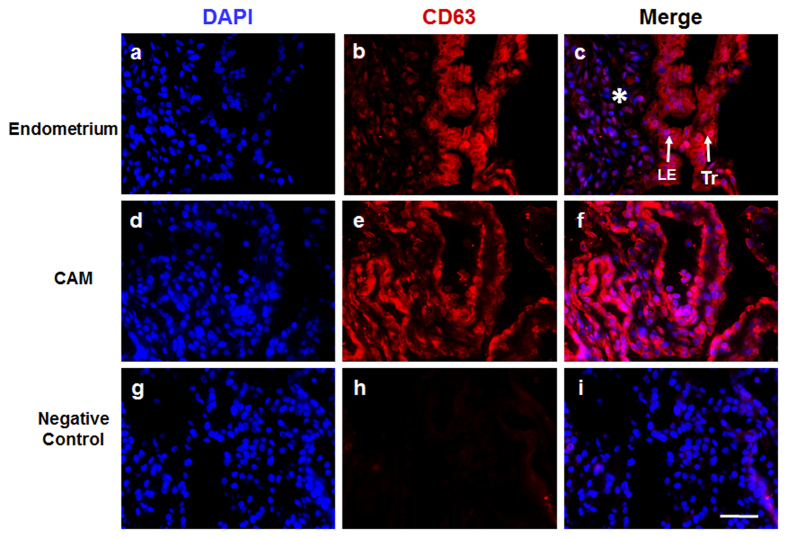
Porcine endometrium and CAM expresses CD63, a well characterized exosome marker. CD63 immunoflourescence on formalin-fixed, paraffin-embedded porcine endometrial (**a–c**) and CAM (**d–f**) biopsies isolated at day 20 of gestation. Nuclei are stained with DAPI (blue; **a**,**d**,**g**), CD63 is stained with Anti-Rabbit CD63, reactive against pig (Red; **b**,**e**,**h**) followed by merge (**c**,**f**,**i**) to demonstrate its localization. *Endometrial stroma; LE: luminal epithelial layer of the uterus; Tr: Trophoblast, Magnification: 400x.

We performed CD63 immunoflourescence on formalin-fixed and paraffin-embedded porcine endometrial (Fig. 3a–c) and CAM (Fig. 3d–f) biopsies obtained at day 20 of gestation. CD63 localized in the cytoplasm/cell membrane of CAM, indicating that placenta may secrete EVs. After demonstrating the presence of CD63 expression *in vivo*, we further investigated whether PTr2 cells express CD63 *in vitro* using immunocytochemistry. Our results confirm the expression of CD63 protein by the PTr2 cells (Supplementary Fig. S2).

### PTr2 and PAOEC derived extracellular vesicles carry miRNA cargo

**Figure 4:**
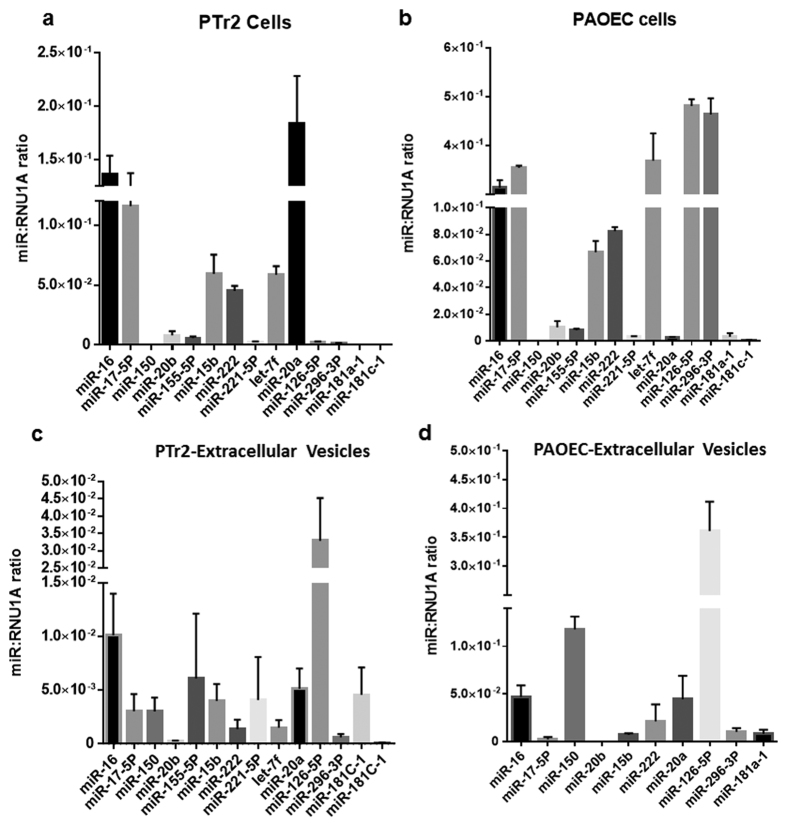
PTr2 and PAOEC derived EVs contain miRNAs. (**a**) Real-time PCR confirmed the expression of literature curated 14 miRNAs that are involved in the angiogenesis regulation. In PTr2 cells, miR-16, miR-17-5P, miR-15b, let-7f, and miR-20a were relatively abundant. (**b**) PAOEC cells also expressed all miRNAs. Among these, miR-16, miR-17-5P, let-7f, miR-126-5P, and miR-296-5P were relatively abundant. (**c**) PTr2 derived EVs contain all 14 miRNAs; however, only miR-126-5P was relatively abundant compared to all other miRNAs. (**d**) PAOEC derived EVs only contained 10 out of 14 miRNAs while miR-126-5P being relatively abundant. miR-155-5P, miR-221-5P, let-7f, and miR-181c-1 were either absent or not detectable in the samples. Relative levels of miRNA expression normalized to RNU1A levels and data are presented as mean ± SEM. Data is derived from three independent experiments; n = 5.

To investigate the possibility of presence of miRNAs in the EVs released by the PTr2 and PAOEC cells, we tested the presence of 14 select miRNAs that regulate angiogenesis and other physiological processes associated with placental development. PTr2 expressed all 14 miRNAs and miR-16, miR-17-5P, miR-15b, let-7f, and miR-20a were found in greater abundance (Fig. 4a). We also found that PAOEC express all miRNAs. Among these, miR-16, miR-17-5P, let-7f, miR-126-5P, and miR-296-5P were relatively abundant (Fig. 4b). After establishing the presence of miRNAs in the parent cells, we investigated whether EVs released by these cell types contain the same miRNAs in a proportional concentration. PTr2 derived EVs contain all 14 miRNAs; however, only miR-126-5P was relatively abundant compared to all other miRNAs (Fig. 4c). PAOEC derived EVs only contained 10 out of 14 miRNAs, with miR-126-5P being relatively abundant. miR-155-5P, miR-221-5P, let-7f, and miR-181c-1 were either absent or not detectable in our samples (Fig. 4d).

### Proteomic analysis of PTr2 and PAOEC derived extracellular vesicles

**Figure 5:**
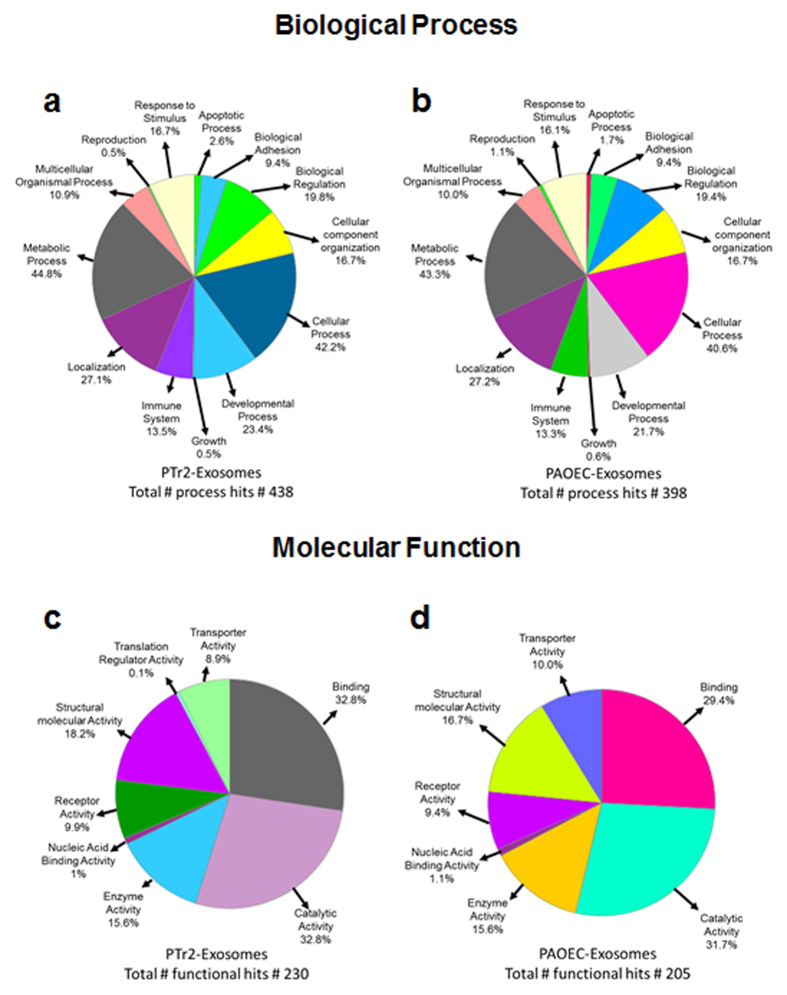
Analysis of PTr2 and PAOEC derived-EV proteins identified by mass spectrometry using PANTHER software. Extracellular vesicle proteins isolated from PTr2 and PAOEC cell supernatants were subjected to ontology and pathway analysis using PANTHER and Gene ontology algorithms and subsequently classified based on their (**a**) Biological Process and (**b**) Molecular function.

LCMS-MS/MS identified an average of 187 proteins in PTr2 derived EVs and 150 proteins in PAOEC derived EVs (Supplementary Data S1). The list of peptides from both PTr2 and PAOEC were subjected to gene ontology and pathway analysis using PANTHER and Gene ontology algorithms and subsequently classified based on biological process (PTr2: Fig. 5a and PAOEC: Fig. 5b) and molecular function (PTr2: Fig. 5c and PAOEC: Fig. 5d). In the biological process, the most clusters identified in PTr2 EVs were: cellular component organization, cellular process, developmental process, metabolic process and protein localization on cell membrane (Fig. 5a). In PAOEC derived EVs, similar clusters were identified but the enrichment was varied (Fig. 5b). Molecular functions of proteins enriched in PTr2 EVs including binding, catalytic activity, enzymatic activity, receptor and transport activity (Fig. 5c). Proteins present in PAOEC EVs were involved in similar activities as PTr2 EVs but the levels of enrichment were different (Fig. 5d). Finally, PANTHER pathway analysis provided potential pathways that are regulated by the proteins present in EVs (Supplementary Fig. S3). Out of these, the 23 most relevant pathways from PTr2 EV group as well as PAOEC EV group were chosen in order to determine the similarity between the functions of EVs secreted by two different cell types. Out of 10 common pathways, angiogenesis, VEGF signaling, inflammation pathway mediated by chemokines, T and B cell activation, Wnt and integrin signaling were the prominent pathways that are regulated by the proteins that are enriched in both PTr2 and PAOEC EVs.

### *In vitro* model of bidirectional trophoblast-endothelial cell communication

**Figure 6:**
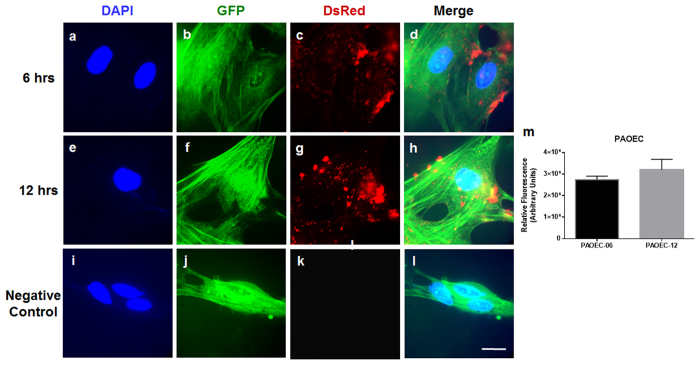
*In vitro* model of trophoblast-endothelial cell communication. (**a–l**) *In vitro* transfer system using PTr2 cells are donors and PAOEC cells as recipients. Extracellular vesicles were isolated from culture supernatants of PTr2 cells. PTr2 derived fluorescently labelled EVs (20 μg/mL) were then added to PAOECs grown in a 6-well cell culture plate and allowed to incubate at 37 °C for 6 hrs (**a–d**) and 12 hrs (**e–h**) in a two set of experiments. PTr2 derived EVs were successfully internalized by the endothelial cells in a time dependent manner. Relative fluorescence emitted by the EVs was calculated in order to measure the concentration of EV uptake. There was a slight increase in the relative fluorescence indicating the increased uptake over a period of time (**m**). Nuclei are stained with DAPI (blue; **a**,**e**,**i**), cytoplasm is stained with CellTracker^TM^ Green BODIFY^®^ dye (Green; **b**,**f**,**j**), EVs were labelled with CM-Dil (CellTracker, C7000; Red; **c**,**g**,**k**) and followed by merge (**d**,**h**,**l**) to demonstrate their localization in the cells. Control wells received the same preparation (PBS+DMSO+CM-Dil) except EVs in triplicate. Data is derived from three independent experiments.

To demonstrate the functional miRNA containing EV transfer between trophoblasts and endothelial cells, we employed an *in vitro* model system using PTr2 and PAOEC. To confirm the possibility of this system serving as an *in vitro* model of cell-to-cell communication via EVs, we first examined the transfer of PTr2 derived EVs to PAOECs in a time dependent manner (Fig. 6). We treated the PAOECs grown in a 6-well cell culture plate in triplicates with a 20 μg/mL of fluorescently labelled PTr2-derived EVs and allowed it to incubate at 37 °C for 6 hrs (Fig. 6a–d; see also Supplementary Video S1 for detailed 3D view of EV uptake). Time dependent uptake of EVs was determined by incubating the culture dish for 12 hrs. (Fig. 6e–h; see also Supplementary Video S2 for detailed 3D view of EV uptake). PTr2 derived EVs were successfully internalized by the endothelial cells in a time dependent manner. Relative fluorescence emitted by the EVs was used to calculate the concentration of EV uptake (Fig. 6m). Our data suggest that there was a slight increase in the relative fluorescence indicating an increased uptake over a period of time.

**Figure 7:**
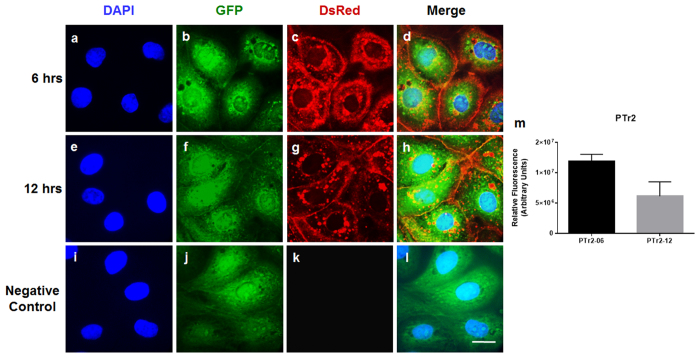
*In vitro* model of endothelial-trophoblast cell communication. (**a–l**) *In vitro* uptake of PAOEC derived EVs by PTr2 cells in a time dependent manner. PAOEC derived fluorescently labelled EVs (20 μg/mL) were then added to PTr2 cells grown in a 6-well cell culture plate and allowed to incubate at 37 °C for 6 hrs (**a–d**) and 12 hrs (**e–h**) in a two set of experiments. PTr2 cells were able to successfully uptake the PAOEC derived EVs in a time dependent manner. Concentration of EVs uptake was measured by calculating the relative fluorescence emitted by the PAOEC derived EVs. Slight decrease in the relative fluorescence was observed, indicating the disappearance of EVs in PTr2 cells over a period of time (**m**). Nuclei are stained with DAPI (blue; **a**,**e**,**i**), cytoplasm is stained with CellTracker^TM^ Green BODIFY^®^ dye (Green; **b**,**f**,**j**), EVs were labelled with CM-Dil (CellTracker, C7000; Red; **c**,**g**,**k**) and followed by merge (**d**,**h**,**l**) to demonstrate their localization in the cells. Control wells received the same preparation (PBS+DMSO+CM-Dil) except EVs in triplicate. Data is derived from three independent experiments.

To test whether our observation with PAOECs occurs in reverse direction, we treated the PTr2 cells with a 20 μg/mL of fluorescently labelled PAOEC-derived EVs and allowed it to incubate at 37 °C for 6 hrs (Fig. 7a–d; see also Supplementary Video S3 for detailed 3D view of EV uptake) and 12 hrs (Fig. 7e–h; see also Supplementary Video S4 for detailed 3D view of EV uptake) as previously described. As expected, PTr2 cells were able to internalize PAOEC derived EVs. However, when we compared the concentration of EV uptake between 6 hrs and 12 hrs, there was a slight decrease in the relative fluorescence indicating the disappearance of EVs in PTr2 cells over a period of time (Fig. 7m). Further we tested whether PTr2 derived EVs can be taken up by PTr2 cells (Supplementary Fig. S4). Similarly, PAOEC derived EVs can be taken up by PAOEC cells using the same conditions (Supplementary Fig. S5). Both the cell types did not take up EVs as much as they were when we used vice-versa conditions.

### PTr2 derived extracellular vesicles promote endothelial cell proliferation

**Figure 8:**
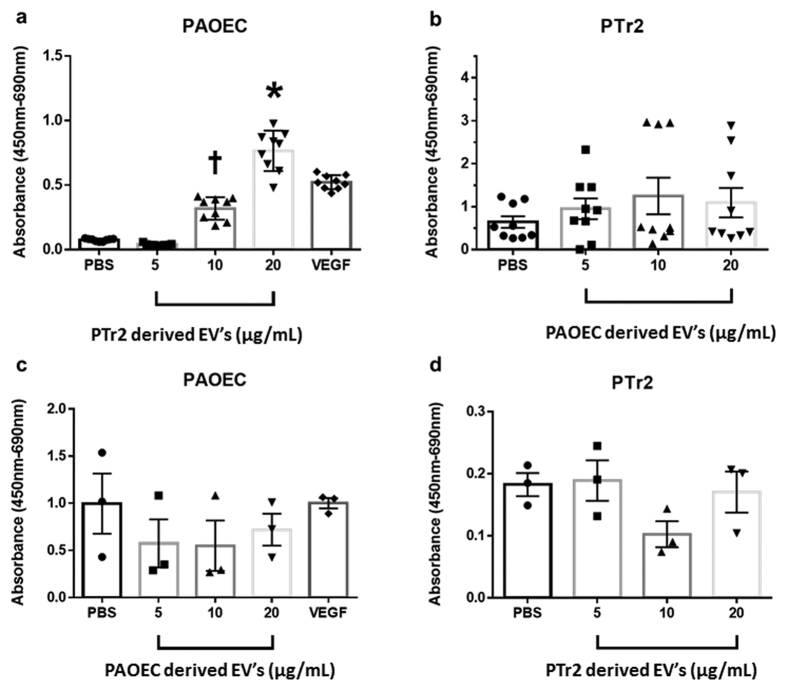
Effect of extracellular vesicles on cell proliferation. (**a**) PTr2 derived EVs promote endothelial cell proliferation. PTr2 derived EVs in different concentrations (5, 10 or 20 μg protein/mL) were added to PAOECs grown in a 6-well cell culture plate and allowed to incubate at 37 °C for 24 hrs. PTr2 derived EVs significantly increased PAOEC proliferation in a dose-dependent manner (p < 0.05, n = 5). PAOECs treated with 10 μg/mL and 20 μg/mL of PTr2 derived EVs had significantly higher proliferation compared to other treatments. (**b**) PTr2 cells grown in cell culture plate were treated with PAOEC derived EVs in different concentrations (5, 10 or 20 μg protein/mL) and allowed to incubate at 37 °C for 24 hrs. PAOEC derived EVs had no significant effect on PTr2 cell proliferation. Similarly, (**c**) PAOEC derived EVs (5, 10 or 20 μg protein/mL) were added to PAOECs grown in a culture plate and allowed to incubate at 37 °C for 24 hrs. PAOEC derived EVs did not have significant effect on PAOEC cell proliferation. Finally, (**d**) PTr2 derived EVs did not have significant effect on PTr2 cell proliferation after 24 hrs of incubation at 37 °C. *p < 0.05, Data is presented as mean ± SEM and derived from three independent experiments.

WST-1 cell proliferation assays were used as a standard endpoint to assess the proliferative effect of PTr2 derived EVs. We treated PAOECs grown in a 6-well cell culture plate with different concentrations (5, 10 or 20 μg protein/mL) of PTr2 derived EVs and allowed it to incubate at 37 °C for 24 hrs. PTr2 derived EVs significantly increased PAOEC proliferation in a dose-dependent manner (p < 0.05, n = 5; Fig. 8a). PAOECs treated with 20 μg/mL of PTr2 derived EVs had significantly higher proliferation compared to PAOECs that were treated with 5 and 10 μg/mL of EVs (p < 0.05). Similarly, PAOECs treated with 10 μg/mL of PTr2 derived EVs had significantly higher proliferation compared to PAOECs that were treated with 5 μg/mL of EVs (p < 0.05).

We then investigated the effect of PAOEC derived EVs on PTr2 cell proliferation at different concentrations (5, 10 or 20 μg protein/mL). PAOEC derived EVs at different concentrations had no significant effect on the proliferation of PTr2 cells (p > 0.05, n = 5; Fig. 8b). When PAOEC cells were treated with PAOEC derived EVs (Fig. 8c) and PTr2 cells with PTr2 EVs (Fig. 8d) there was considerable variability in the absorbance levels between control PBS group and EV treated groups.

## Discussion

Recognition and maintenance of pregnancy depends on coordinated and precisely regulated cellular and molecular processes resulting in endometrial growth, conceptus development, implantation, and placenta formation that are accompanied by the establishment of unique conceptus-endometrial cross talk^24^. Recently, EVs have been shown to be vesicles of great potential that can participate in and facilitate embryo-uterine cross talk. Transfer of genetic material and proteins between cells at a distant site via exosomes/microvesicles is a relatively new concept for cell-cell communication^36^. Therefore, we aimed to characterize and investigate the role of EVs secreted by porcine endometrium and CAM/placenta *in vivo* as well as PAOEC and PTr2 cells *in vitro*. The current study sheds new light on the physiological relevance of secretory genomic information by EVs, and hints that EVs to be potentially new mediators of intercellular signaling during embryo-uterine dialogue.

Here, for the first time, we (1) identified that EVs are present in the endometrium and CAM as well as in the cell supernatants of PAOECs and PTr2 cell lines; (2) investigated the validity of these EVs by the presence of known surface marker, CD63 and size analysis; (3) showed that EVs contain miRNAs that can potentially regulate angiogenesis which is an important process in peri-attachment period; (4) we further characterized signaling molecules (proteins) present in the EVs; (5) importantly, demonstrated that EVs derived from PTr2 can be shuttled into PAOEC and vice versa; (6) Finally, examined the effects of EVs derived from PTr2 on PAOEC cell proliferation and *vice versa*.

A classical approach to any experiment seeking to elucidate the physiological or pathophysiological role of EVs is their specific isolation and characterization. Methods have already been established to accurately characterize EVs in any biological tissues or fluids^37^. Those methods are primarily based on presence of known surface markers and particle size. According to classification, exosomes are nanovesicles with a diameter of 30–100 nm and express characteristic membrane surface markers such as CD9, CD63 and CD81 along with heat shock proteins HSP70 and HSP90. Many studies involving humans^5^, mice^38^, and pigs^39^, have confirmed the presence of exosomes in various reproductive tissues. Very relevant to our study, we wanted to investigate the presence of EVs in the endometrium and CAM. As a first approach, using TEM, we were able to confirm the presence of EVs in the extracellular space of endometrial tissues and in the close vicinity of chorionic villi. Due to unavailability of pig specific exosome markers such as CD9 and CD81, testing CD63 expression in our tissues was the only feasible option. Nevertheless, both the endometrium (luminal epithelial layer) and the CAM expressed CD63, both intracellularly and along the cell surface. CD63 is primarily expressed on monocytes, macrophages, and activated platelets, and is also weakly expressed on granulocytes, T cell and B cells. However, using immunocytochemistry we confirmed the specific expression of CD63 in the representative porcine placental cell line, PTr2. Due to the focus of our investigation on feto-maternal cross-talk, we decided to use two representative cell lines, PTr2 (representing the fetal side) and PAOEC (representing the endometrial vasculature). We isolated EVs from the cell supernatants of PTr2 and PAOEC using a modified differential centrifugation and miRCURY exosome isolation kits. This method provided us with sufficient EVs to use in all downstream applications. Using TEM, we confirmed the negatively stained EVs. Particle size analysis revealed PTr2 and PAOEC cell derived EV pellet consisting mostly exosomes; however, few traces of microvesicles (100–200 nm) were found. Immunogold labelling of the PTr2 derived EVs specifically identified CD63 molecule on the characteristic exosomal membrane. Finally, the detection of CD63 protein in the cells and their EVs by Western blot analysis supports and confirms our other findings in the present study.

Recent studies involving cell-cell communication via exosomes have mostly focused on the content and their exchange between the distant cells to aid in transfer of signals^34^. Exosomes are now known to contain lipids, proteins, and numerous RNA species including mRNA and miRNAs. These biomolecules are shown to transfer from the cell of origin to adjacent or distant cells and influence biological functions of the recipient cells. Since Valadi *et al*. 2007 demonstrated the presence of miRNAs in exosomes, there has been growing interest in the role of exosomal miRNAs in cell-cell communication. We sought to determine whether selected pro- and anti-angiogenic miRNAs involved in modulation of angiogenesis at the maternal-fetal interface are present in the representative cell lines (PTr2 and PAOCs) and in EVs derived from them. As anticipated, pro- and anti-angiogenic miRNAs were expressed in the PAOEC cells but the same set of miRNAs were also present in the PTr2 cells. In addition, EVs released by PTr2 also contained these miRNAs including the relatively abundant pro-angiogenic miR-126-5P. These findings suggest that EVs potentially participate in the regulation of placental angiogenesis. However, a few miRNAs such as miR-155, miR-221, let-7f, miR-181C-1 were not detected in the EVs released by PAOEC and we are unable to speculate about their absence.

Similar to miRNAs, protein trafficking between cells can influence functions of recipient cells. The type and concentration of protein present in the exosome is highly dependent on the cell type and pre-conditioning^18^. One of the first studies to characterize the exosomal proteome was from mesothelioma cells, in which only 38 proteins of various types were identified. Studies involving isolation of exosomes from a human first trimester trophoblast cell line^40^ and placental mesenchymal stem cells^23^ identified numerous proteins that are involved in various cellular and molecular processes including: immune system regulation, reproduction, apoptosis, biological adhesion, cellular binding, receptor and enzymatic activity. Our EV proteome study identified various signaling pathways including angiogenesis and cellular communication that are relevant to early events during porcine pregnancy. Overall, our study provides the first proteomic analysis of EVs derived from PTr2 and PAOEC cells, shedding light on the influence of PTr2 derived EVs on placental angiogenesis and endometrial vasculature.

After investigating the content and potential functions that may be regulated by EVs, we evaluated whether miRNAs and protein loaded EVs can be transferred to recipient cells. In order to test this hypothesis, we established an *in vitro* model of bidirectional trophoblast-endothelial cell communication. The biological processes in the recipient cells are influenced by exosomes released by the cell of origin and its surrounding conditions. Currently, exosomes are thought to fuse with the plasma membrane of the recipient cell and release their contents into the target cell. Previous studies have proposed a mechanism that there is binding at the cell surface via surface receptors^41^, or that internalization of exosomes is by endocytosis^42^. In our study, PTr2 derived EVs were successfully internalized by PAOEC and vice versa. In order to test the specificity of EV uptake, we treated PTr2 cells with PTr2 derived EVs and PAOEC cells with PAOEC derived EVs. The recipient cells did not take up the EVs originated by the same cell supporting the idea that EVs derived from one cell type are more likely taken up by distant cells of different phenotype. However, we did not perform any experiments to determine specificity of EV uptake either by PTR2 or PAOC cells.

Finally, we assessed the effect of EVs on the recipient cells and their functions. We treated the PAOECs with EVs derived from PTr2 and *vice versa*. PTr2 derived EVs were able to stimulate the PAOEC proliferation *in vitro*. When we treated PAOEC cells with PAOEC derived EVs and PTr2 cells with PTr2 derived EVs, we found considerable variability in the control PBS groups compared to EV treated groups. This discrepancy could be attributed to the quality of cells as well as potential variability in the numbers of cells used in this experiment. Nevertheless, this experiment did not conclusively establish whether EVs of the same cell origin have any effect on the parent cell. Further experiments are needed to establish the specificity of PTr2 or PAOEC derived EVs on biological functions.

Importantly, these findings support the idea that EVs containing miRNA and proteins can elicit a biological response in the recipient cells leading to increased proliferation which is crucial during peri-implantation period. We were, however, unable to assign specific miRNAs or proteins involved in potentiation of proliferation. Future studies should be conducted by labelling specific miRNAs based on their abundance in exosomes to determine specific function.

In summary, this study supports the notion that EVs are present at the porcine maternal-fetal interface, contain specific miRNAs and several important proteins, and are capable of successfully delivering their cargo *in vitro*. Additionally, identification of specific miRNAs and proteins have enabled bioinformatic identification of pathways that could be influenced if the EVs are taken up by the endothelial cells or trophectoderm at the time of implantation. Taken together, this study provides critical insight into cell-cell communication at the porcine maternal-fetal interface that aids in successful conceptus-endometrial cross talk.

## Materials and Methods

### Endometrial and chorioallantoic membrane tissue collection

This study was approved and conducted in accordance with the recommendation by the Animal Care Committee of the University of Guelph (Animal Utilization Protocol Number 10R061). Endometrial and chorioallantoic membrane (CAM) tissues were collected as previously described^43^. Briefly, the reproductive tracts were collected immediately after euthanizing sows on gestation day 20 at the University of Guelph abattoir. All conceptuses were exposed for visual examination. Endometrial and CAM tissues were collected and formalin fixed and paraffin embedded for downstream applications. Similarly, tissues were also collected for transmission electron microscopy.

### Cell culture

Porcine trophectoderm cells (PTr2) were kindly provided by Dr. Laurie A. Jaeger, College of Veterinary Medicine, Purdue University. PTr2 cells are established from dispersed cell culture of day 12, filamentous conceptus obtained from pigs. These cells have been previously characterized for SN1/38 positive, cytokeratin positive, vimentin negative, express fibronectin and many of the integrin subunits present in porcine trophectoderm *in vivo*. These also express ER alpha and PR via immunocytochemistry. Detailed isolation methods have been published^44^. Briefly, PTr2 cells were maintained in special medium containing DMEM-F12 (21041-025), Phenol Red-free, charcoal stripped sFBS (5%; F6765, Sigma-Aldrich, St. Louis, MO, USA), bovine insulin (0.1 Units/mL; 10516; Sigma-Aldrich, St. Louis, MO, USA), glutamine (1X; i.e., 2 mM), Penicillin-streptomycin (1X). Porcine aortic endothelial cells (PAOEC) were purchased from the Cell Applications, Inc., San Diego, CA, USA. PAOEC’s were maintained according to manufacturer’s instructions using the porcine endothelial cell medium (P211-500) and subcultured using subculture reagent kit (090 K; Cell Applications, Inc., San Diego, CA, USA). Supernatant from both the PTr2 and PAOEC cultures was collected for exosome isolation.

### Isolation of extracellular vesicles from culture supernatants

Extracellular vesicles were isolated from the supernatant of both the PTr2 and PAOEC cell lines according to the combination of methods from Thery *et al*.^37^ and using miRCURY exosome isolation kit (300102; Exiqon, inc. Woburn, MA, USA). Briefly, culture supernatants were harvested and sequentially centrifuged at 4 °C at 300 × g for 10 min, 2000 × g for 10 min, and 10 000 × g for 30 min. This serial centrifugation step was used to eliminate cells, dead cells, and cell debris. The supernatants were filtered through a 0.22 μm filter and subsequently ultracentrifuged at 100 000 × g for 70 min at 4 °C and the top 2/3 portion of supernatant was discarded. The remaining 1/3^rd^ portion was pooled and 2 mL of precipitation buffer was added to every 10 mL of supernatant and allowed to incubate overnight at 4 °C. The supernatant was centrifuged at 20 °C at 3200 × g for 30 min to pellet EVs. The EV pellet was resuspended in 200 μL of resuspension buffer. The total protein concentration of purified EVs was determined using a Pierce BCA protein assay kit (23225; Life Technologies Inc. Burlington, ON, Canada).

### Transmission electron microscopy on endometrial and fetal tissues

To assess the presence of EVs in the endometrium and CAM, standard TEM procedure was used. Briefly, tissues were dehydrated in alcohol, embedded in epoxy resin, ultrasectioned, transferred to 300-mesh Formvar coated nickel grids (Electron Microscopy Sciences) and stained using 4% uranyl acetate and lead citrate according to previously published methods^45^. The grids were subsequently observed using a JEM-1010 transmission electron microscope (Jeol, Korea) at 100 kV. Images were obtained and processed using Gatan Microscopy Suite (GMS) 3 Software (Gatan, Inc. Pleasanton, CA, USA).

### Immunoelectron microscopy analysis of extracellular vesicles

Extracellular vesicles isolated from PTr2 and PAOEC supernatants were mixed with equal quantities of freshly prepared 4% paraformaldehyde for 20 min. Samples were washed using PBS, pelleted by ultracentrifugation and then fixed in 2% glutaraldehyde for 5 min. After fixation, EVs were resuspended in PBS and 5 μL of the sample was transferred to 300-mesh Formvar coated nickel grids. The sample was allowed to settle by incubating at room temperature (RT) for 20 min. Grids were stained using 4% saturated aqueous uranyl acetate and lead citrate for approximately 10 min. The prepared grids were examined under a Hitachi H-7000 transmission electron microscope system (Hitachi High-Technologies) operated at 80 kV. Extracellular vesicles can be quantified using three available standard methods: (1) Transmission Electron microscopy; (2) nanotracking (nanoparticle) analysis using nanosight; (3) ExoELISA kit (System Biosciences Inc), which are specific to quantify exosomes/microvesicles. We chose transmission electron microscopy due to its easy access. Randomly selected regions (at least 6) were analyzed for the presence of EVs, photographed and individual EV size were measured using ImageJ software^46^,^47^ by adjusting the scale to TEM image.

Immunogold labelling of CD63 was performed using a rabbit polyclonal IgG (sc-15363; Santa Cruz Biotechnology, Inc. Dallas, TX, USA) reactive against pigs. Extracellular vesicle samples were fixed using 4% paraformaldehyde as previously described and loaded onto 300-mesh Formvar coated nickel grids and allowed to settle for 20 min at RT. Extracellular vesicle bearing grids were blocked with 5% bovine serum albumin in PBS and subsequently incubated with anti-CD63 for 30 min at 37 °C. The grids were then incubated with goat anti-rabbit CD63 immunoglobulin G, pAb conjugated to 12-nm colloidal gold particles (H&L EM grade; Jackson ImmunoResearch laboratories, Inc. West Grove, PA, USA). Immunolabelled EVs were postfixed with 2% glutaraldehyde in PBS for 30 min at RT. The grids were negatively stained with 4% uranyl acetate. Control grids were treated with PBS instead of primary antibody and samples were examined under a Hitachi H-7000 TEM. Extracellular vesicles visualized under microscope were subjected to morphometric analysis. The size and specificity of the CD63 positive EVs were performed on electron micrographs using ImageJ software (National Institute of Health, Bethesda, MD, USA).

### Western blot analysis

Total protein was isolated from PTr2 and PAOEC cells as well as their secreted EVs using Total exosome RNA and protein isolation kit (#4478545; Life Technologies Inc. Burlington, ON, Canada). Briefly, exosomal pellets as well as freshly harvested cells were treated with 100–625 μL of ice-cold resuspension buffer. The cell lysate was vortexed and passed through 26 gauze needle and incubated on ice for 5–10 minutes to ensure complete cell lysis. Micro BCA protein assay kit (#23235; ThermoFisher Scientific, Burlington, ON, Canada) was used to measure the protein concentration. Equal amount of protein sample was loaded and separated on 4–20% pre-cast mini-PROTEAN TGX gels (Bio-Rad Laboratories, Mississauga, ON, Canada) and run at 140 V for 1 h. The proteins were transferred onto PVDF membranes (Bio-Rad Laboratories, Mississauga, ON, Canada). Blots were incubated at 4 °C overnight with anti-pig CD63 rabbit polyclonal IgG at a concentration of 1:100. This was followed by incubation with secondary antibody (Goat anti-rabbit IgG H&L (HRP), ab97051, 1:10000). Electro-chemiluminescence detection was performed using Bio-Rad Immun-Star Clarity Chemiluminescent kit (Bio-Rad Laboratories, Mississauga, ON, Canada). The X-ray films were developed and analyzed using Image J software (NIH, Bethesda, MD, USA) to obtain densitometry values.

### Total RNA extraction and purification

Total RNA including miRNA from PTr2 and PAOEC cells was extracted using Total RNA extraction kit (Norgen BioTek Corp, Thorold, ON, Canada). Total RNA within EVs was isolated using Total exosome RNA and protein isolation kit (#4478545; Life Technologies Inc. Burlington, ON, Canada) according to manufacturer’s instructions. The concentration and purity of isolated total RNA was assessed using a Nanodrop 2000 C UV-Vis Spectrophotometer (Thermo Scientific, Wilmington, DE, USA) and stored at −80 °C until further use.

### Real-time PCR analysis

Real-time PCR for selected miRNAs was carried out using Qiagen miRNA Assays in a LightCycler real-time PCR system (LightCycler, Roche Diagnostics, Laval, QC, Canada) according to manufacturer’s instructions. Briefly, miRNAs present in the total RNA samples were reverse transcribed using miscript II RT kit (Qiagen, Mississauga, ON, Canada). The resultant cDNA was assessed for the concentration and purity using Nanodrop 2000 C UV-Vis Spectrophotometer (Thermo Scientific, Wilmington, DE, USA) and stored at −20 °C until required. miRNA expression relative to RNU1A reference genes. RNU1A is wet bench validated for PCR assays and its stable expression in the porcine tissues has been documented previously^48^. The focus of this investigation was to study the miRNAs that are involved in regulating the angiogenesis and therefore we conducted an extensive literature search which allowed us to list out 14 miRNAs for further investigation. The select miRNAs include: miR-16, miR-17-5P, miR-150, miR-20b, miR-155-5P, miR-15b, miR-222, miR-221-5P, let-7f, miR-20a, miR-126-5P, miR-296-3P, miR-181a-1, miR-181c-1. Porcine specific primer assays for selected miRNAs were custom designed using sequences available in the miRBase database^49^,^50^,^51^ version 19 (http://www.mirbase.org) and ordered from Qiagen (Qiagen, Mississauga, ON, Canada). The mature miRNA ID and accession numbers are listed in Supplementary Table S1. A melt curve analysis was conducted as an additional quality control after running each PCR plate.

### Immunoflourescence

Formalin-fixed and paraffin-embedded porcine endometrial and CAM biopsies obtained at day 20 of gestation were sectioned (5 μm) and mounted on glass slides. CD63 immunoflourescence was performed as per the standard immunofluorescence protocol. Briefly, sections were deparaffinized, rehydrated, CD63 antigen was retrieved using 10 mM sodium citrate pH 6.0 at 95 °C for 30 min. Sections were blocked using 2% bovine serum albumin and then slides were incubated with primary antibody (CD63; rabbit polyclonal IgG; sc-15363; Santa Cruz Biotechnology, Inc. Dallas, TX, USA) overnight at 4 °C. Secondary antibody, donkey anti-rabbit IgG H&L (Alexafluor^®^ 594; ab150076; 1:10000) was applied and incubated for 1 hr at room temperature in the dark. After washing with PBS, slides were mounted with Prolong^®^ Gold Antifade Reagent with DAPI (#8961; Thermofisher Scientific, Inc. Carlsbad, CA, USA). The slides were examined by epifluorescence microscopy and photographed with an AxioCam-equipped M1 imager (Zeiss, Toronto, ON, Canada) with Axiovision 4.8 software.

### Immunocytochemistry

PTr2 cells were grown on coverslips for 24 hrs, fixed with 100% methanol (chilled at −20 °C) at RT for 5 min followed by ice-cold PBS wash three times for 5 min. Samples were permeabilized using PBS containing 0.1% Triton X-100 and washed with PBS three times for 5 min. Cells were blocked using 1% BSA, 22.52 mg/mL glycine in PBST (PBS+ 0.1% Tween 20) for 30 min and then incubated with diluted primary antibody (1:100; CD63, rabbit polyclonal IgG; sc-15363; Santa Cruz Biotechnology, Inc. Dallas, TX, USA) in 1% BSA in PBST in a humidified chamber overnight at 4 °C. Cells were washed with PBS and secondary antibody was applied at a concentration of 1:10000. Slides were mounted with Prolong^®^ Gold Antifade Reagent with DAPI and observed and photographed using epifluorescence microscopy and Axiovision 4.8 software.

### Proteomic analysis of extracellular vesicles by mass spectrometry

Total protein from PAOEC and PTr2 cell derived EVs was extracted using Total exosome protein isolation kit (#4478545; Life Technologies Inc. Burlington, ON, Canada) according to manufacturer’s instructions. The sample was desalted with a dialysis in 10 mM ammonium bicarbonate solution and subsequently digested with trypsin (20 μg) at 37 °C for 18 h. The peptide mixture was further analyzed by Liquid Chromotagraphy (LC)/Mass Spectrometry (MS) LC-MS/MS on a nLC-Orbitrap Velos Pro (Thermo Scientific) and MS/MS spectra were collected. The data were searched using Thermo Proteome Discoverer 1.4.0.288.

### Extracellular vesicle proteome analysis

MS/MS identified proteins were analyzed by PANTHER (Protein Analysis Through Evolutionary Relationships; http://www.pantherdb.org). This software specialized in predictions and classification of proteins in order to facilitate high-throughput analysis. Using this software, the classified proteins were further classified according to their biological and molecular function. Protein IDs were also subjected to further classification using available protein database as a background. This process yielded families and subfamilies of proteins that were annotated with ontology terms and sequences were assigned to PANTHER pathways, cell signaling pathways.

### Shuttling assays using confocal microscopy

Shuttling and internalization of EVs was performed using confocal microscopy. Briefly, freshly harvested EVs derived from either PAOEC or PTr2 were stained with CM-Dil (CellTracker, C7000), a fluorescent dye that labels the plasma membrane, according to the manufacturer’s instructions. Next, fluorescently-labelled EVs were diluted in respective medium and were added into either PTr2 or PAOEC cell cultures grown on coverslips in 6 well cell culture plates. At the indicated time points, cells were fixed with 100% methanol (chilled at −20 °C) at RT for 5 min followed by ice-cold PBS wash three times for 5 min. CellTracker^TM^ Green BODIFY^®^ dye (1:1000) was added to the cells and allowed to incubate at 37 °C for 30 min. Cells were briefly washed and coverslips were mounted with Prolong^®^ Gold Antifade Reagent with DAPI. After 2 mins, the coverslips were examined under a confocal microscope and images were acquired for further processing ImageJ software.

### Cell proliferation assays

Cell proliferation assay was performed as previously described^52^. Briefly, PAOEC and PTr2 cells were cultured at the rate of 1.25 × 10^4^ cells/well in two separate 96 well plates for 24 hrs. Each well was treated with varying concentrations of EVs derived from either PTr2 or PAOEC (5, 10, and 20 μg/mL) in triplicates with PBS as control. After treating the wells with EV preparations, the rest of the wells were filled with medium to make up the volume to 100 μL. Plates were allowed to incubate in humidified chamber at 37 °C for 24 hours. 10 μL of WST-1 reagent was added to each well and incubated for 2 hrs at 37 °C. Absorbance in each plate was measured using microtitre plate reader at 450–690 nm with the reference wavelength of ~650 nm.

### Statistical Analysis

miRNA PCR data was analyzed using the ∆∆Ct method; gene expression levels of each miRNA was measured against RNU1A as a reference gene. Data are presented as mean ± SEM (n = 5 for each miRNA expression analysis). Comparison between multiple groups in the cell proliferation data was performed by means of analysis of variance (ANOVA) using GraphPad Prism 6.01 (GraphPad Software, La Jolla, CA, USA). If ANOVA demonstrated a significant interaction between variables, post hoc analyses were performed by the multiple-comparison Bonferroni correction test. P ≤ 0.05 value between groups was considered to be statistically significant. All experiments were conducted three times and in triplicate (i.e. biological replicates).

## Additional Information

**How to cite this article**: Bidarimath, M. *et al*. Extracellular vesicle mediated intercellular communication at the porcine maternal-fetal interface: A new paradigm for conceptus-endometrial cross-talk. *Sci. Rep.* **7**, 40476; doi: 10.1038/srep40476 (2017).

**Publisher's note:** Springer Nature remains neutral with regard to jurisdictional claims in published maps and institutional affiliations.

## Supplementary Material

Supplementary Information

Supplementary Video S1

Supplementary Video S2

Supplementary Video S3

Supplementary Video S4

Supplementary Dataset S1
