# Supplementary material for: Extracellular vesicle mediated intercellular communication at the porcine maternal-fetal interface: A new paradigm for conceptus-endometrial cross-talk
Source: Sci Rep. 2017 Jan 12;7:40476. doi: 10.1038/srep40476 (PMC5228034; doi:10.1038/srep40476)
Supplement: Supplementary Information [file srep40476-s1.pdf]

Supplementary Information:

Extracellular vesicle mediated intercellular communication at the porcine maternal-fetal interface: A new paradigm for conceptus-endometrial cross-talk

Mallikarjun Bidarimath[¶], Kasra Khalaj[¶†], Rami T. Kridli[†§], Frederick W. K. Kan[¶], Madhuri Koti[¶], and Chandrakant Tayade[¶†*]

[¶]Department of Biomedical and Molecular Sciences, Queen's University, Kingston, Ontario, Canada, K7L 3N6; and [†]Department of Biomedical Sciences, Ontario Veterinary College, University of Guelph, Guelph, Ontario, Canada, N1G 2W1. [§]Department of Animal Production, Faculty of Agriculture, Jordan University of Science and Technology, Irbid, 22110, Jordan.

[*]Corresponding Author

Supplementary Figures

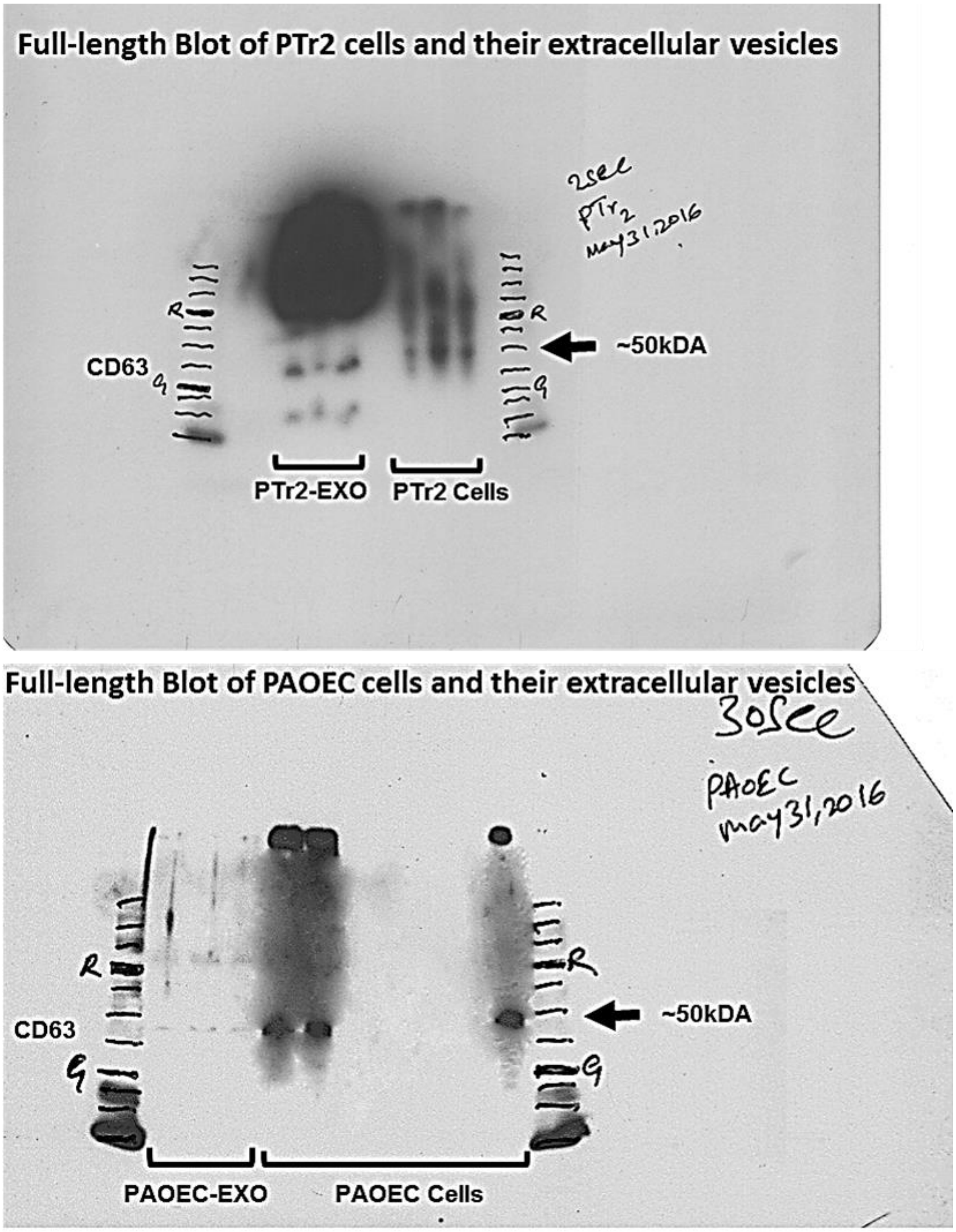

Supplementary Figure S1. Full-length blots of Western blots performed on PTr2 cells and PTr2-derived EVs as well as PAOEC cells and PAOEC-derived EVs.

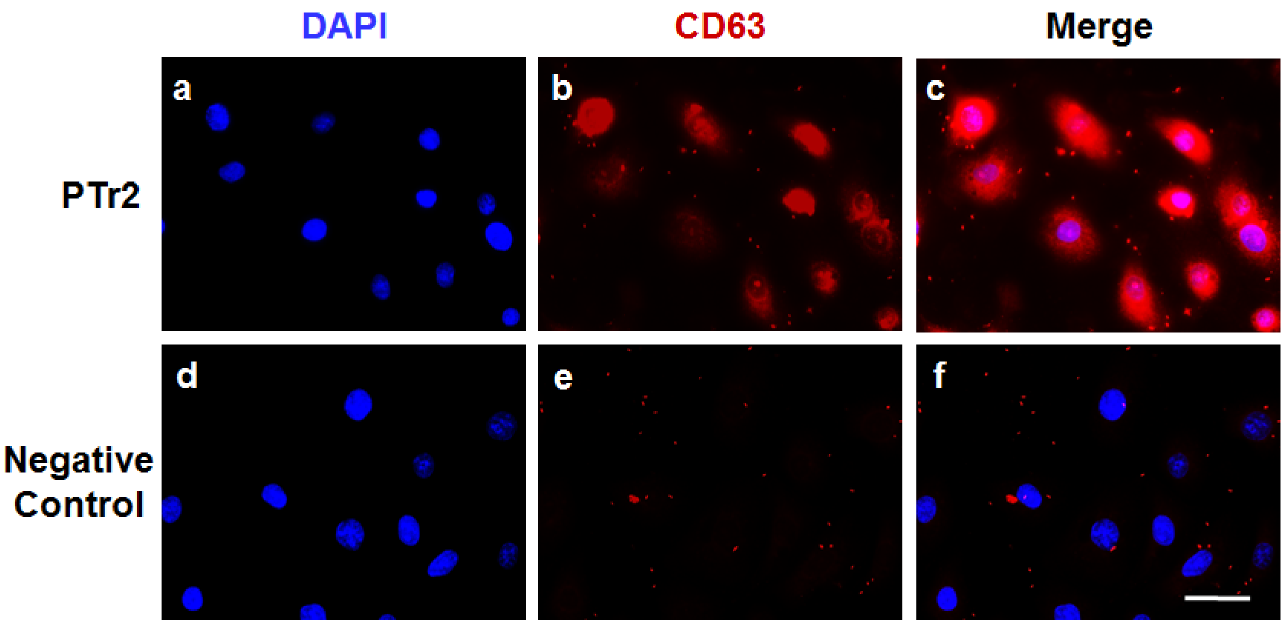

Supplementary Figure S2. CD63 immunocytochemistry on PTr2 cells *in vitro*. a-c) PTr2 cells are established from dispersed cell culture of day 12 conceptus obtained from pigs. Fluorescence microscopic analysis revealed CD63 protein expression in the cytoplasm/cell membrane of PTr2 cells (c). Nuclei are stained with DAPI (blue; a and d), CD63 is stained with Anti-Rabbit CD63, reactive against pig (Red; b and e) followed by merge (c and f) to demonstrate its localization. Magnification: 400X.

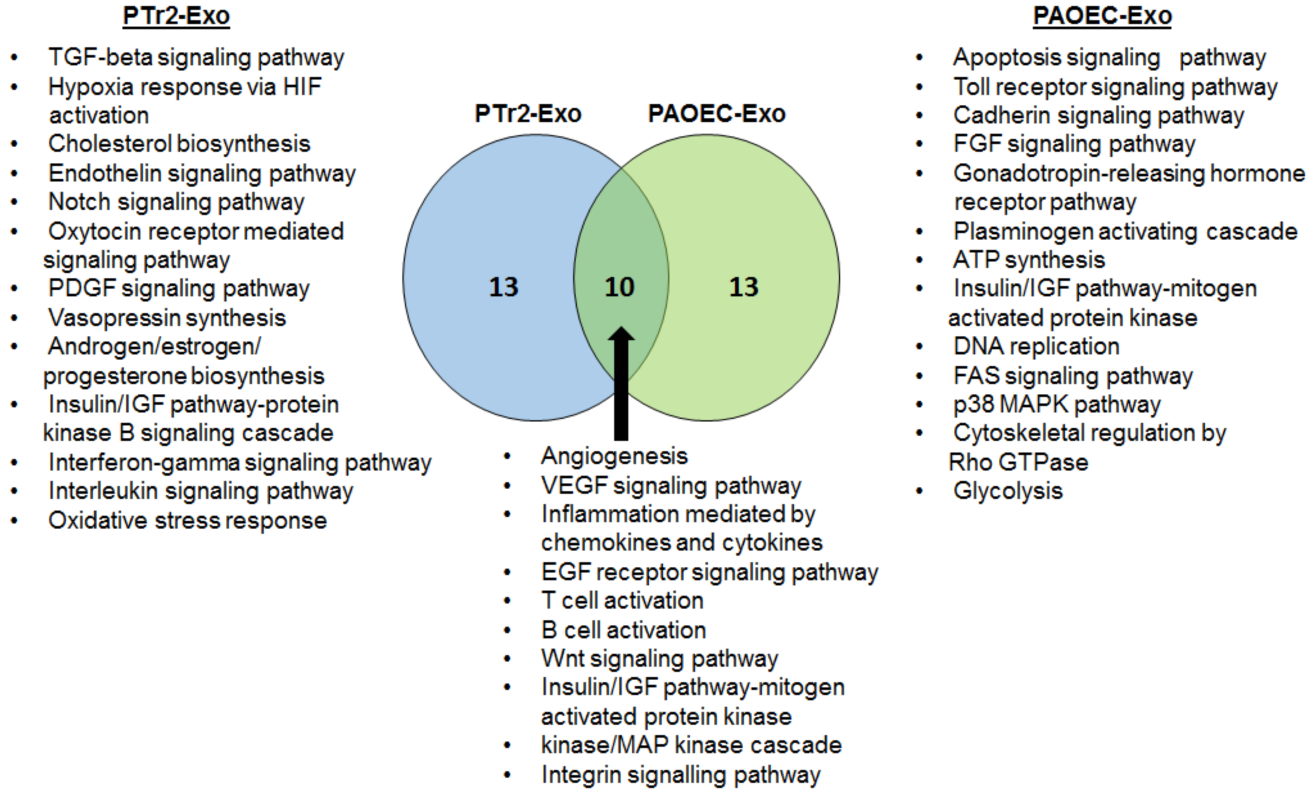

Supplementary Figure S3. PANTHER pathway analysis of PTr2 and PAOEC derived EV proteins. Bioinformatic pathway analysis provided many potential pathways that are regulated by the proteins present in the EVs. Out of these, 23 most relevant pathways from each group were chosen to further study the similarity between the functions elicited by EVs originated from representative cells of both the maternal and the fetal side. The Venn diagram depicts the distribution of common and unique EVs protein regulated signalling pathways relevant to early pregnancy in pigs. Out of 23 pathways, 10 signalling pathways were common and appear to be regulated by EVs proteins derived two different cell types.

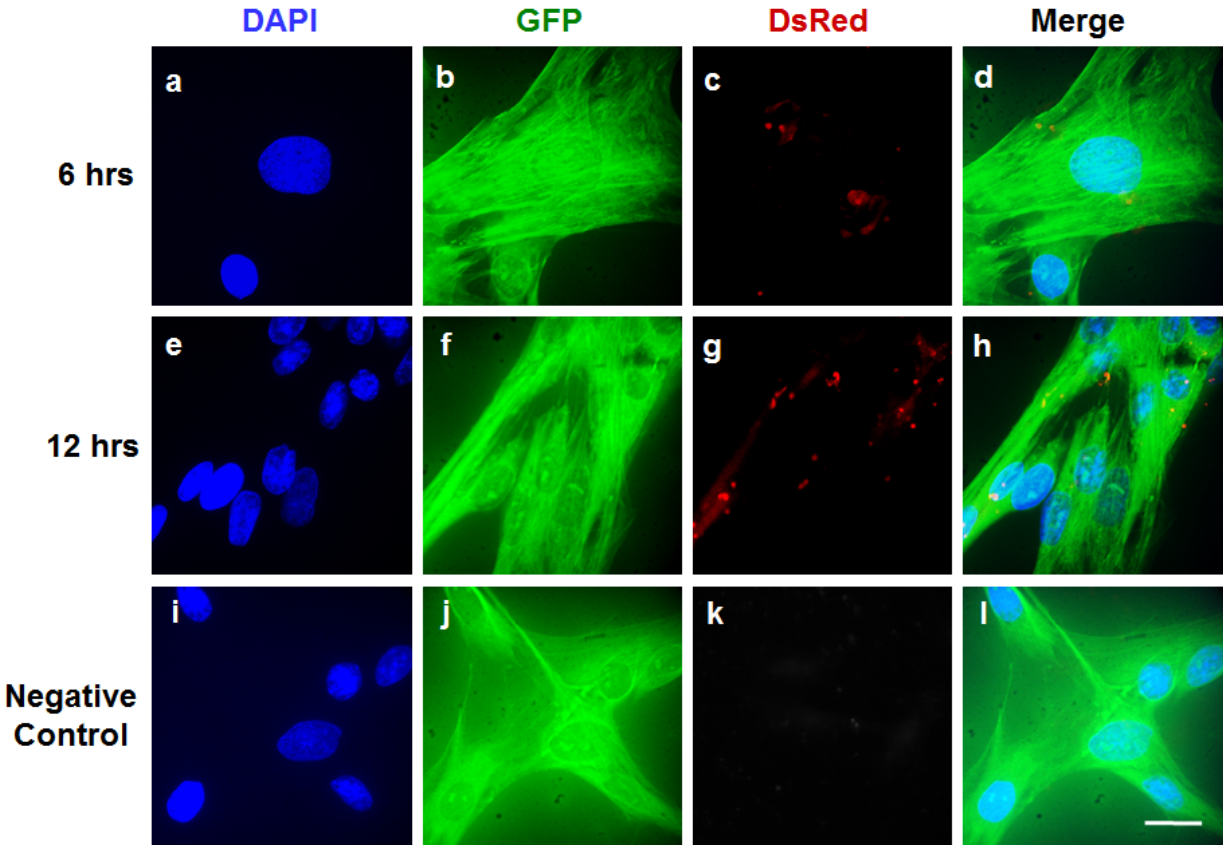

Supplementary Figure S4. *In vitro* uptake of PAOEC derived EVs by PAOEC cells in a time dependent manner. PAOEC derived fluorescently labelled EVs (20 µg/mL) were then added to PAOEC cells grown in a 6-well cell culture plate and allowed to incubate at 37°C for 6 hrs (a-d) and 12 hrs (e-h) in a two set of experiments. PAOEC cells were able to uptake the PAOEC derived EVs. The uptake was not pronounced at 6 hrs time point but at traces of PAOEC derived EVs can be noticed at 12 hrs time point. Nuclei are stained with DAPI (blue; a, e and i), cytoplasm is stained with CellTracker™ Green BODIFY® dye (Green; b, f and j), PAOEC derived EVs were labelled with CM-Dil (CellTracker, C7000; Red; c, g and k) and followed by merge (d, h and l) to demonstrate their localization in the cells. Data is derived from three independent experiments.

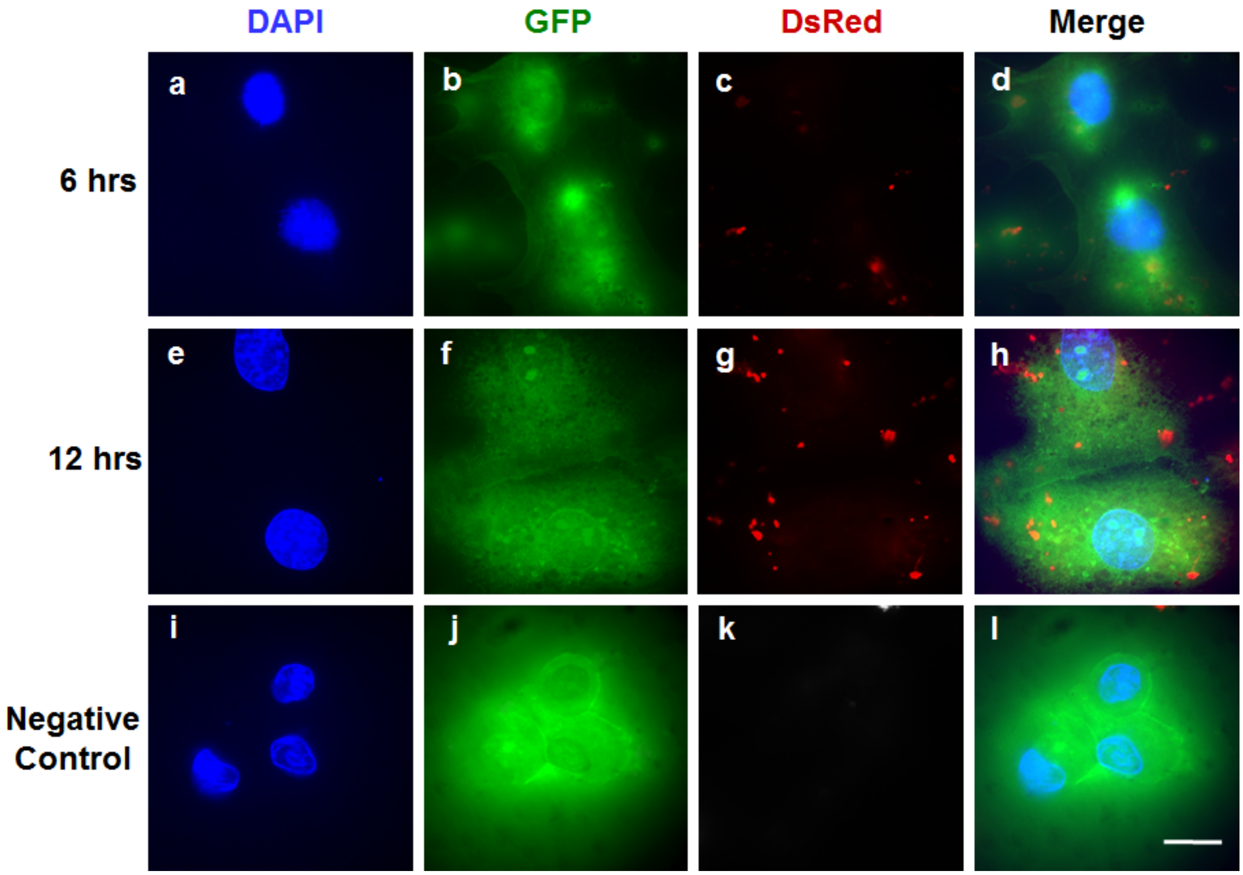

Supplementary Figure S5. In vitro transfer of PTr2 derived EVs to PTr2 cells grown in a 6-well cell culture plate. Fluorescently labelled PTr2 derived EVs (20 µg/mL) were added to PTr2 cells and allowed to incubate at 37°C for 6 hrs (a-d) and 12 hrs (e-h) in a two set of experiments. At 6 hrs time point, only traces of labelled EVs were taken up by PTr2 and at 12 hrs the the amount of EVs uptake was increased. Nuclei are stained with DAPI (blue; a, e and i), cytoplasm is stained with CellTracker™ Green BODIFY® dye (Green; b, f and j), PTr2 derived EVs were labelled with CM-Dil (CellTracker, C7000; Red; c, g and k) and followed by merge (d, h and l) to demonstrate their localization in the cells. Data is derived from three independent experiments.

Supplementary Table:

Supplemental Table S1. List of validated primers of selected mature miRNA ID, miRBase Accession numbers, and mature miRNA Sequences used for real time PCR Analysis.

MATURE MIRNA ID	MIRBASE ACCESSION NO.	MATURE miRNA SEQUENCE
ssc-miR-16	MIMAT0007754	5'UAGCAGCACGUAAAUAUUGGCG
ssc-miR-17-5p	MIMAT0007755	5'CAAAGUGCUUACAGUGCAGGUAG
ssc-miR-150	MIMAT0025365	5'UCUCCCAACCCUUGUACCAGUG
ssc-miR-20b	MIMAT0025359	5'CAAAGUGCUCACAGUGCAGGUAG
ssc-miR-155-5p	MIMAT0022959	5'UUAAUGCUAAUUGUGAUAGGGG
ssc-miR-15b-5P	MIMAT0002125	5'UAGCAGCACAUCAUGGUUUACA
ssc-miR-222	MIMAT0013942	5'AGCUACAUCUGGCUACUGGGUCUC
ssc-miR-221-5p	MIMAT0022949	5'ACCUGGCAUACAAUGUAGAUUUCUGU
ssc-let-7f-5p	MIMAT0002152	5'UGAGGUAGUAGAUUGUAUAGUU
ssc-miR-20a-5P	MIMAT0002129	5'UAAAGUGCUUAUAGUGCAGGUA
ssc-miR-126-5p	MIMAT0018377	5'CAUUAUUACUUUUGGUACGCG
ssc-miR-296-5p	MIMAT0017952	5'GAGGGCCCCCCCAAUCCUGU
Ssc-miR-181a-1	MIMAT0010191	5'AACAUUCAACGCUGUCGGUGAGUU
Ssc-miR-181c-1	MIMAT0002144	5'AACAUUCAACCUGUCGGUGAGU
RNU1A	ENSG00000207389	5'AAAAAGGGCTTCTGTCGTGA

Supplementary Videos:

Supplementary Video S1. In vitro transfer system using PTr2 cells as donors and PAOEC cells as recipients after 6hrs.

Supplementary Video S2. In vitro transfer system using PTr2 cells as donors and PAOEC cells as recipients after 12 hrs.

Supplementary Video S3. In vitro uptake of PAOEC derived EVs by PTr2 cells after 6hrs.

Supplementary Video S4. In vitro uptake of PAOEC derived EVs by PTr2 cells after 12hrs.

Supplementary Data:

Supplementary Data S1. List of proteins identified in the PTr2 and PAOEC derived EVs using mass spectrometry. Total protein was extracted from PTr2 (n = 3) and PAOEC (n = 3) derived EVs and subjected to LCMS-MS/MS to identify different peptides present in the samples. Mass spectrometry identified an average of 187 proteins in PTr2 derived EVs and 150 proteins in PAOEC derived EVs. This list of proteins from both PTr2 and PAOEC derived EVs was subjected to PANTHER Pathway analysis. Pathway analysis revealed 172 potential signaling pathways in which PTr2 derived EVs proteins are involved. Similarly, PAOEC derived EVs protein pathway analysis revealed 36 potential signaling pathways.
